# Immunotherapy in advanced kidney cancer: an alternative meta-analytic method using reconstructed survival data in case of proportional hazard assumption violation

**DOI:** 10.3389/fonc.2022.955894

**Published:** 2022-09-05

**Authors:** Luigi Nocera, Giuseppe Fallara, Daniele Raggi, Federico Belladelli, Daniele Robesti, Francesco Montorsi, Pierre I. Karakiewicz, Bernard Malavaud, Guillaume Ploussard, Andrea Necchi, Alberto Martini

**Affiliations:** ^1^ Division of Experimental Oncology/Unit of Urology, URI, Urological Research Institute, Istituto di Ricovero e Cura a Carattere Scientifico (IRCCS) San Raffaele Scientific Institute, Milan, Italy; ^2^ Department of Oncology, Istituto di Ricovero e Cura a Carattere Scientifico San Raffaele Scientific Institute, Milan, Italy; ^3^ Cancer Prognostics and Health Outcomes Unit, Division of Urology, University of Montreal Health Center, Montreal, QC, Canada; ^4^ Department of Urology, Institut Universitaire du Cancer Toulouse - Oncopôle, Toulouse, France; ^5^ Department of Urology, La Croix du Sud Hospital, Toulouse, France

**Keywords:** metastatic renal cell carcinoma (mRCC), overall survival (OS), progression-free survival (PFS), duration of response (DoR), individual patient data (IPD), reconstructed data

## Abstract

**Background:**

With the advent of immuno-oncology compounds in randomized trials, we observe more and more survival curves crossing. From a statistical standpoint this corresponds to violation of the proportional hazard assumption. When this occurs, the hazard ratio from the Cox regression is not reliable as an estimate. Herein, we aimed to identify the most appropriate IO-based therapy for metastatic renal cell carcinoma applying an alternative method to overcome the issue of hazard assumption violation for meta-analyses.

**Methods:**

Pubmed, EMBASE, Web of Science and Scopus databases were searched. Only phase III randomized clinical trials on IO-IO (nivo-ipi) or IO-TKI combinations were included. An algorithm to obtain survival data from published Kaplan-Meier curves was used to reconstruct data on overall survival (OS), progression-free survival (PFS) and duration of response (DoR). Differences in restricted mean survival time (RMST) were used for comparisons.

**Results:**

individual survival data from 4,206 patients from five trials were reconciled. Patients who received nivo-ipi or IO-TKI had better OS, PFS and DoR relative to sunitinib (all p<0.001). Patients who received IO-TKI had similar OS and PFS relative to nivo-ipi, with a 36-month ΔRMST of -0.55 (95% CI: -1.71-0.60; p=0.3) and -1.5 (95% CI: -2.9-0.0; p=0.051) months, respectively. Regarding DoR, patients who received nivo-ipi had longer duration of response relative to IO-TKI, with a 24-month ΔRMST of 1.5 (95% CI: 0.2-2.8; p=0.02) months.

**Conclusion:**

Despite overall similar OS and PFS for patients receiving nivo-ipi and IO-TKI combinations, DoR was more favorable in patients who received nivo-ipi compared to IO-TKI. A meta-analysis based on differences in RMST is a useful alternative whenever the proportional hazard assumption is violated.

**Systematic Review Registration:** https://www.crd.york.ac.uk/prospero/, identifier CRD42021241421.

## Introduction

Sunitinib, a receptor tyrosine kinase inhibitor (TKI), represents a standard-of-care therapeutic option in first-line setting of advanced clear-cell renal cell carcinoma (mRCC) since several years ago (1). More recently, increasing evidence supporting the use of immune-oncology (IO) compounds (2–4), and consequently the results from pivotal randomized clinical trials (RCT) with IO-based combinations, played a major role in shifting the therapeutic landscape of mRCC upfront therapy towards these combinations. Nivolumab plus ipilimumab (5, 6), pembrolizumab plus axitinib (7, 8), avelumab plus axitinib (9, 10), nivolumab plus cabozantinib (11, 12) and atezolizumab plus bevacizumab (13) received approval by the United States Food and Drug Administration (US-FDA) as first-line therapy for mRCC. Additionally, lenvatinib plus pembrolizumab showed compelling results in phase III CLEAR trial which randomized patients to receive this combination versus lenvatinib plus everolimus versus sunitinib (14).

The introduction of different IO-based strategies, similar in major outcomes, has raised the important clinical need to elucidate which are the key differences in the efficacy spectrum among therapies, in order to tailor treatment selection and patient counselling. A comparison among therapies is quite challenging, due to the lack of a prospective randomized comparison, thus determining potentially significant biases among study populations. When trying to make a comparison from published data, traditional measures such as median survival or hazard ratios may underestimate the potential benefit of therapies characterized by response proportions unable to affect the median and/or by atypical response kinetics. To address this void, we performed a meta-analysis using reconstructed survival data derived from phase III RCTs and a network meta-analysis to indirectly compare overall survival (OS), progression-free survival (PFS) and duration of response (DoR) in IO-IO versus IO-TKI combinations, using an alternative, yet more appropriate, statistical method: restricted mean survival time (RMST).

## Evidence acquisition

### Methodology

We performed a systematic review and meta-analysis of phase III RCTs comparing IO-IO and/or IO-TKI combinations relative to sunitinib in the setting of treatment-naïve mRCC. Notably, the study IMmotion151 (13), which focused on atezolizumab plus bevacizumab was not included to minimize the heterogeneity of addressed therapies. Indeed, bevacizumab is a monoclonal antibody that uniquely targets the vascular endothelial growth factor receptor, thus having different pharmacodynamics than TKIs. Similarly, the lenvatinib plus everolimus arm of the CLEAR trial (14) was excluded, everolimus being a selective inhibitor of mTOR (mammalian Target Of Rapamycin).

We considered only English language RCTs, while observational studies, review articles, commentaries, editorials and articles without peer-review were excluded. Bibliographies were hand-searched for completeness. Meeting abstracts of medical societies were also searched. In case of more than one publication addressing the same RCT, we focused on the most recent one. The review was conducted according to the Preferred Reporting Items for Systematic Reviews and Meta-Analyses (PRISMA) guidelines (15).

### Outcome measures

The primary outcome was to compare OS, PFS and DoR among patients who received IO-TKI relative to IO-IO combinations. All outcomes were assessed using RMST up to 12, 24 and 36 months of follow-up. RMST is a measure of survival time to time of occurrence of a specific outcome, and can be interpreted as the area under the survival curve, *i.e.* the integral of the survival function from time zero up to a time point:


$$\left(\int_{t_{0}}^{t_{t}}S\right)$$

### Search strategy

The protocol for this systematic review and meta-analysis was registered in PROSPERO (registration number: CRD42021241421). PubMed, EMBASE, Web of Science, Scopus databases were searched for studies indexed from January 1, 2016 to March 20, 2021. The following keywords were used to identify potential reports: (renal cell carcinoma OR kidney cancer OR renal cancer) AND (metastatic or advanced) AND [(systemic OR first-line) AND (therapy OR treatment)] AND (RCT OR randomized clinical trial). References from commentaries, editorials, conference publications, review articles, and from included studies were hand-searched and cross-referenced for completeness. Conference abstracts reporting unpublished data were included. Titles and abstracts of manuscripts were used to screen for initial study inclusion. Full text review was performed when the abstract was insufficient to determine study inclusion.

### Eligibility and data extraction

Given our statistical strategy of reconstructing survival data based on Kaplan-Meier (KM) curves, only RCTs including KM curves for OS, PFS and DoR were eligible. Survival data reconstruction consisted of utilizing published KM curves to indirectly extract reconstructed survival data on survival and follow-up through a digital reconstruction of figures (16, 17). More in detail, we digitally scanned KM curves from included RCTs and reconstructed survival data using an algorithm that derives individual survival data from digitized published KM curves by measuring curve drops relative to the number of patients at risk and events (18).

### Study review methodology and risk of bias assessment

To optimize methodological quality, two authors completed the study selection independently (L.N. and A.M.), according to PRISMA assessment. Disagreements were resolved by consensus with all co-authors. Risk of bias was determined using The Cochrane Collaboration’s tool, which assesses selection, performance, detection, attrition and reporting bias, and other sources of bias (19).

### Statistical analysis

RMST up to 18, 24 and 36 months for OS, PFS and DoR in patients treated with IO-IO or IO-TKI combinations was estimated employing a reconstructed survival data approach to obtain pooled survival probability curves. RMSTs and associated 95% confidence intervals (CI) up to 18, 24 and 36 months were derived from the quantification of the area under the survival probability curve through the trapezoidal rule.

To support the use of RMST instead of traditional hazard ratios, we tested the assumption of proportionality of hazards on the reconstructed datasets, using the Grambsch and Therneau test (20).

OS, PFS and DoR were compared between IO-IO and IO-TKI combinations. Therefore, reconstructed data were grouped based on the category of treatment received: sunitinib, IO-TKI or IO-IO. Being 18 months the common maximum follow-up time across all treatment groups, we estimated 18-month RMST to perform comparisons of outcomes over the maximum common study period (21). Moreover, 24- and 36-month RMSTs were also assessed, considering that a considerable proportion of patients in each treatment group reached longer follow-up times. Indirect comparisons between IO-TKI and IO-IO combinations were also made, using a network meta-analysis approach (22).

Finally, a sensitivity analysis was performed in the intermediate/poor risk population, using reconstructed survival data extracted from KM curves. Being nivolumab plus ipilimumab (nivo-ipi) the major first-line option for these patients, it was compared with the other treatments for which KM curves were reported in the intermediate/poor risk groups.

All analyses were performed using the R statistical package v3.6.1 (R Project for Statistical Computing) (23) and STATA 14 (StataCorp LP, College Station, TX, USA). All tests were two-sided, with a significance level set at *p*<0.05.

## Evidence synthesis

### Literature search results

**Figure 1:**
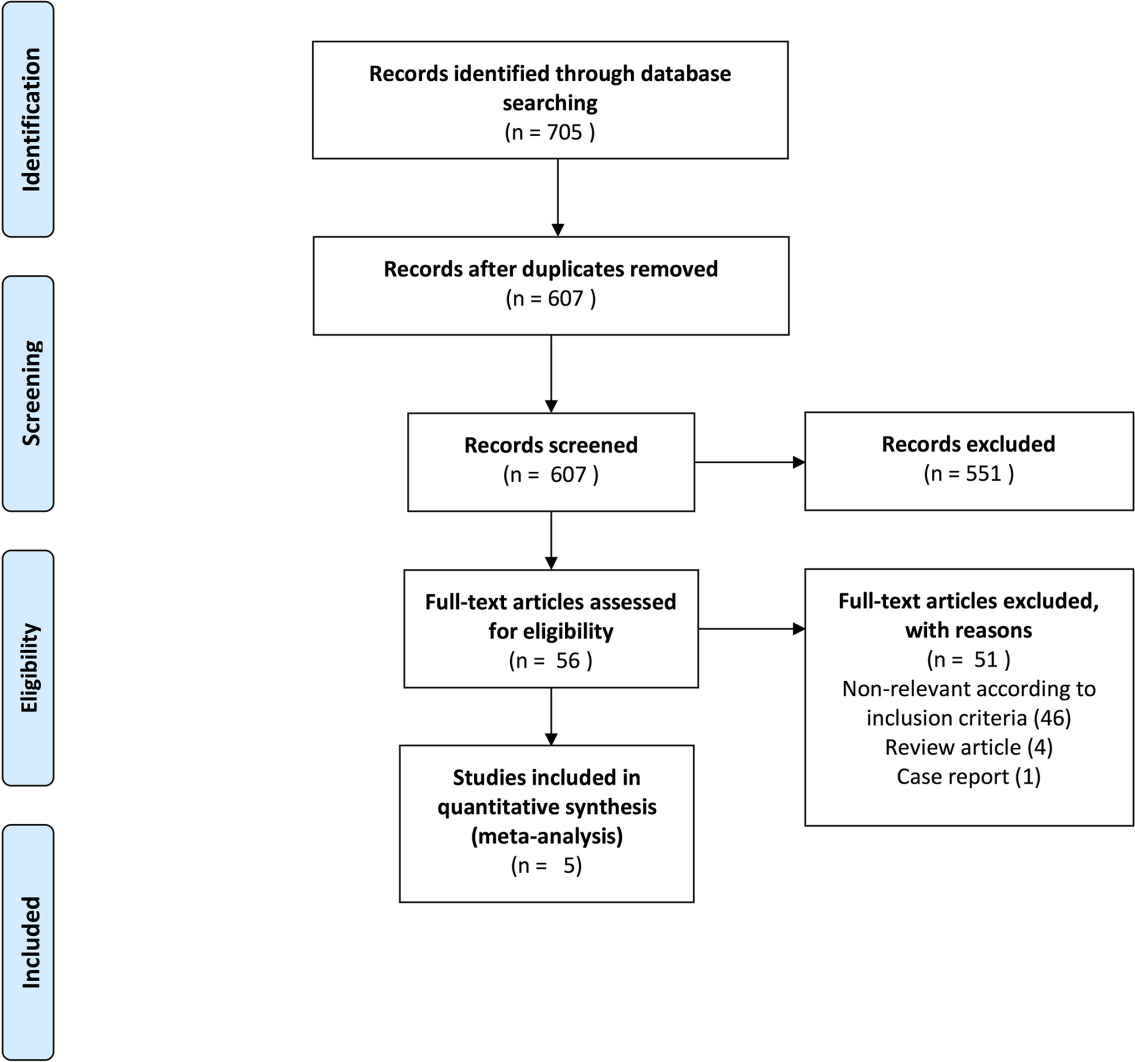
PRISMA (Preferred Reporting Items for Systematic Reviews and Meta-Analyses) flow chart depicting included studies for the meta-analysis addressing overall survival, progression-free survival and duration of response of first-line therapy in mRCC patients.

Through electronic search, we identified 705 publications and selected 56 potential studies following abstract screening. After full-text review, we identified five first-line RCTs examining IO-IO or IO-TKI combinations relative to sunitinib, in the setting of treatment-naïve mRCC, **Figure 1**.

### Characteristics of included trials

CheckMate 214 (5, 6, 24), JAVELIN Renal 101 (9, 10), KEYNOTE-426 (7, 8), KEYNOTE-581 (14) and CheckMate 9ER (11, 12) studies were included in the meta-analysis (**Table 1**). Specifically, the first focused on the IO-IO combination nivolumab plus ipilimumab (nivo-ipi), while the others examined IO-TKI combinations, i.e. avelumab plus axitinib, pembrolizumab plus axitinib, lenvatinib plus pembrolizumab and nivolumab plus cabozantinib, respectively. All RCTs were phase III and enrolled treatment-naïve patients with a clear-cell component, starting from 2014 (25). Across all experimental arms of RCTs, median age ranged from 62 to 64 years and most patients were male (from 71 to 77%). Rates of previous nephrectomy ranged from 69% in CheckMate 9ER to 83% in KEYNOTE-426. Finally, the distribution of IMDC risk group was comparable among RCTs, intermediate risk patients being the most common and all RCTs including patients belonging to all groups (favorable, intermediate and poor) (**Table 2**).

**Table 1:** Characteristics of included trials.

Trial	Treatment (N)	Class (IO-IO vs IO-TKI)	Median follow up
CheckMate 214 (ITT)	Nivolumab plus ipilimumab (N=550)	IO-IO	55 months
	Sunitinib (N=546)	TKI	
KEYNOTE-426	Pembrolizumab plus axitinib (N=432)	IO-TKI	31 months
	Sunitinib (N=429)	TKI	
KEYNOTE-581	Lenvatinib plus pembrolizumab (N=355)	IO-TKI	27 months
	Sunitinib (N=357)	TKI	
JAVELIN Renal 101	Avelumab plus axitinib (N=442)	IO-TKI	19 months
	Sunitinib (N=444)	TKI	
CheckMate 9ER	Nivolumab plus cabozantinib (N=323)	IO-TKI	18 months
	Sunitinib (N=328)	TKI	

IO, immune-oncology.

TKI, tyrosine kinase inhibitor.

ITT, intention to treat.

**Table 2:** Baseline characteristics of patients in the experimental arms of included trials.

Characteristics	CheckMate 214 (ITT)	KEYNOTE-426	KEYNOTE-581 (CLEAR)	JAVELIN Renal 101	CheckMate 9ER
Median age, yr	62	62	64	62	62
Male sex, %	75.0	71.0	71.8	71.5	77.1
Nephrectomy, %	82.0	83.0	73.8	79.6	68.7
IMDC risk group, %					
Favorable	23.0	32.0	31.0	21.3	22.9
Intermediate	61.0	55.0	59.2	61.3	58.2
Poor	17.0	13.0	9.3	16.3	18.9
Not reported	–	–	0.6	1.1	–

ITT, intention to treat.

According to the Cochrane Risk of Bias tool, all studies were at low risk of selection (random sequence generation), attrition and reporting bias. Similarly, allocation concealment was not determinable in all RCTs and all were at risk of performance bias. With regard to detection bias, all studies were at low risk, except for CheckMate 214 (**Supplementary Table 1**).

Reconstructed KM curves for each outcome, stratified by trial, are displayed in **Supplementary Figures 1**–**3**. The numbers at risk in the curves demonstrate accurate reconstructed survival data compared to the original studies, allowing for subsequent meta-analysis.

### Overall survival

**Figure 2:**
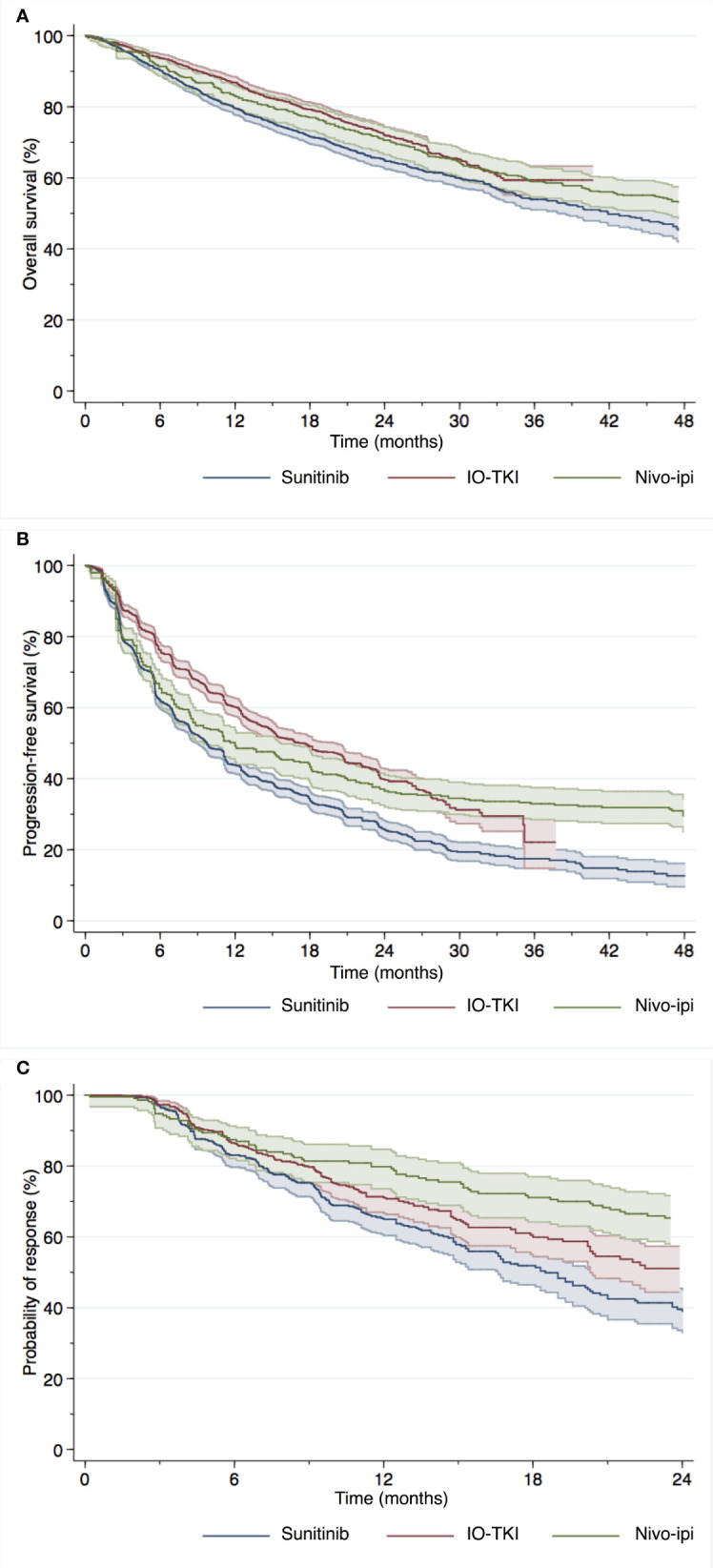
Overall survival **(A)**, progression-free survival **(B)** and duration of response **(C)** of mRCC patients using reconstructed survival data derived from five individual studies with immune-oncology based combination therapy.

KM curves depicting OS of patients enrolled in the included RCTs, divided into treatment groups (sunitinib, IO-TKI and nivo-ipi), using reconstructed data are reported in **Figure 2A**. OS probabilities of patients treated with nivo-ipi at 18, 24 and 36 months were 77%, 71% and 59%, respectively. Similarly, at 18, 24 and 36 months, OS probabilities of patients receiving IO-TKI combinations were respectively 79%, 72% and 59%, while OS probabilities of patients treated with sunitinib were 72%, 65% and 54%, respectively.

When Grambsch and Therneau test was employed on reconstructed datasets from included RCTs, a *p* value of 0.02 was obtained for the comparison between IO-TKI and sunitinib, proving violation of the proportional hazard assumption.

**Table 3** displays the results of the differences in RMST up to the time point of interest. Regarding 18-month RMST, there was a significant difference for patients treated with nivo-ipi versus sunitinib (*p*=0.048). Similarly, there was a significant difference between patients treated with IO-TKI and those receiving sunitinib (*p*<0.001). No significant difference was observed for patients treated with nivo-ipi versus IO-TKI therapy (*p*=0.09). Analogous results were recorded in terms of 24-month RMST. When considering 36-month RMST, there was a significant difference for patients treated with nivo-ipi versus sunitinib (*p*=0.02). Similarly, there was a significant difference between patients treated with IO-TKI and those receiving sunitinib (*p*<0.001). No significant difference was observed for patients treated with nivo-ipi versus IO-TKI therapy (*p*=0.3).

**Table 3:** Differences in restricted mean survival time (delta RMST) up to the time point of interest according to treatment arm.

Arms	ΔRMST up to 18mo of follow-up (95% CI)	ΔRMST up to 24mo of follow-up (95% CI)	ΔRMST up to 36mo of follow-up (95% CI)
	**Overall survival**		
IO-IO vs sunitinib	0.5 (0.0 – 0.9)	0.8 (0.1 – 1.5)	1.4 (0.2 – 2.5)
IO-TKI vs sunitinib	0.8 (0.5 – 1.1)	1.3 (0.8 – 1.7)	1.9 (1.1 – 2.7)
IO-IO vs IO-TKI	-0.4 (-0.8 – 0.1)	-0.5 (-1.2 – 0.2)	-0.6 (-1.7 - 0.6)
	**Progression-free survival**		
IO-IO vs sunitinib	0.8 (0.1 – 1.5)	1.4 (0.5 – 2.3)	3.1 (1.7 – 4.5)
IO-TKI vs sunitinib	2.2 (1.8 – 2.7)	3.1 (2.5 – 3.7)	4.6 (3.6 – 5.6)
IO-IO vs IO-TKI	-1.4 (-2.1 – -0.7)	-1.7 (-2.7 – -0.8)	-1.5 (-2.9 – 0.0)
	**Duration of response**		
IO-IO vs sunitinib	1.5 (0.7 – 2.4)	2.9 (1.6 – 4.2)	
IO-TKI vs sunitinib	0.8 (0.1 – 1.5)	1.5 (0.4 – 2.5)	
IO-IO vs IO-TKI	0.7 (-0.1 – 1.6)	1.5 (0.2 – 2.8)	

RMST for duration of response was calculated only up to 24 months.

### Progression-free survival

KM curves depicting PFS of patients enrolled in the included RCTs, divided into treatment groups (sunitinib, IO-TKI and nivo-ipi), using reconstructed data are reported in **Figure 2B**. PFS probabilities of patients treated with nivo-ipi at 18, 24 and 36 months were 43%, 37% and 33%, respectively. Similarly, at 18, 24 and 36 months, PFS probabilities of patients receiving IO-TKI combinations were respectively 49%, 40% and 22%, while PFS probabilities of patients treated with sunitinib were 34%, 26% and 18% respectively.

When Grambsch and Therneau test was employed on reconstructed datasets from included RCTs, a *p* value less than 0.001 was obtained for the comparisons between both nivo-ipi versus sunitinib and IO-TKI versus sunitinib, proving violation of the proportional hazard assumption.

Regarding 18-month RMST, there was a significant difference for patients treated with IO-IO versus sunitinib (*p*=0.02). Similarly, there was a significant difference between patients treated with IO-TKI and those receiving sunitinib (*p*<0.001). A significant difference was also observed for patients treated with nivo-ipi versus IO-TKI therapy (*p*<0.001). Analogous results were recorded in terms of 24-month RMST. When considering 36-month RMST, there was a significant difference favoring nivo-ipi versus sunitinib (*p*<0.001). Similarly, there was a significant difference between patients treated with IO-TKI and those receiving sunitinib (*p*<0.001). No significant difference was observed between nivo-ipi and IO-TKI therapy (*p*=0.051).

### Duration of response

KM curves with numbers at risk for DoR were available for all included RCTs except for JAVELIN Renal 101, for which reconstructed survival data was not possible. KM curves depicting DoR of patients enrolled in the included RCTs, divided into treatment groups (sunitinib, IO-TKI and nivo-ipi), using reconstructed data are reported in **Figure 2C**. DoR probabilities of patients treated with nivo-ipi at 18 and 24 months were 71% and 65%, respectively. Similarly, at 18 and 24 months, DoR probabilities of patients receiving IO-TKI combinations were respectively 60% and 51%, while DoR probabilities of patients treated with sunitinib were 52% and 39% respectively.

When Grambsch and Therneau test was employed on reconstructed datasets from included RCTs, a *p* value less than 0.001 was obtained for the comparison between both nivo-ipi versus sunitinib and IO-TKI versus sunitinib, proving violation of the proportional hazard assumption.

Regarding 18-month RMST, there was a significant difference for patients treated with nivo-ipi versus sunitinib (*p*<0.001). Similarly, there was a significant difference between patients treated with IO-TKI and those receiving sunitinib (*p*=0.02). No significant difference was observed for patients treated with nivo-ipi versus IO-TKI therapy (*p*=0.09). When considering 24-month RMST, there was a significant difference for patients treated with nivo-ipi versus sunitinib (*p*<0.001). Similarly, there was a significant difference between patients treated with IO-TKI and those receiving sunitinib (*p*=0.007). A significant difference was also observed for patients treated with nivo-ipi versus IO-TKI therapy (*p*=0.02).

### Sensitivity analyses

The only trial including KM curves to derive individual data of patients in the intermediate/poor IMDC category was KEYNOTE-426, which examined pembrolizumab plus axitinib. Sensitivity analyses confirmed better OS and PFS in patients treated with nivo-ipi or pembrolizumab plus axitinib compared to sunitinib (all *p*<0.001; **Supplementary Figures 4**–**5** and **Supplementary Table 2**). No difference was observed in terms of RMST between nivo-ipi and pembrolizumab plus axitinib at all time points where ΔRMST was calculated (**Supplementary Table 2**).

## Discussion

In the context of treatment-naïve mRCC, several combination therapies (IO-IO and IO-TKI) recently joined the available treatment armamentarium. However, it is object of debate which one between IO-IO and IO-TKI combinations represents the best treatment option. Previous meta-analyses have been completed in the setting of treatment-naïve mRCC (26–29). However, no previous study focused on treatment DoR. Moreover, in all reports, the authors used hazard ratios, which may under-/over-estimate the benefit of treatments characterized by atypical response kinetics. Indeed, hazard ratios ignore the time distribution of events during follow-up and Cox proportional hazards assumption is invariably violated upon Kaplan-Meier curves crossing (30). Indeed, nivo-ipi has shown prolonged responses in specific patient subgroups, and data from RCTs have demonstrated a violation of the proportional hazards assumption. To overcome these limitations, alternative statistical methods, such as the RMST, may be used to assess time-dependent outcomes (31, 32). RMST represents a widely accepted, yet unfrequently used, measure that can be interpreted as the average event-free survival time up to a pre-specified, follow-up time (33). It is equivalent to the area under the survival curve from randomization through that time point. RMST difference translates into a gain or loss in event-free survival time, when comparing treatment versus control during the examined period (34–36). No meta-analysis using RMST is currently available in the field of treatment-naïve mRCC.

To address this void, we performed a meta-analysis of reconstructed data from five published RCTs. Our study revealed that both nivo-ipi and IO-TKI combinations showed more favorable OS, PFS and DoR relative to sunitinib. However, no statistically significant difference was observed when comparing these two novel treatment strategies, except for DoR which was more durable in patients treated with nivo-ipi. This could account for the apparently longer PFS observed on the reconstructed Kaplan-Meier curve. Based on the shape of the curves, it might be speculated that the benefit deriving from nivo-ipi tends to manifest later during follow-up and that might be more durable. Yet, longer-term results are needed to verify this hypothesis.

Our study has several methodological strengths, which render it unique in the context of meta-analyses in first-line mRCC treatment. The interpretation of the survival impact of novel therapies in mRCC warrants statistical methods that optimally and faithfully consider treatment effects. Indeed, treatment response kinetics may not be constant due to a non-linear pattern of distribution of events over time. This implies the risk of violation of the proportionality of hazards assumption. Studies examining IO-IO or IO-TKI combinations frequently revealed a prolonged treatment response in a subgroup of patients who survived beyond a specific follow-up time point. This discrepancy in treatment response among patients cannot be ignored due to methodological implications. Indeed, standard methods such as Cox regression analyses, that rely on summary parameters like hazard ratios and/or median survival, may fail to capture the realistic variability of individual treatment response patterns. This intrinsic limitation of hazard ratios, which were at the base of all published meta-analyses in first-line treatment of mRCC, renders previous meta-analyses less robust and accurate, and justifies the need for a novel approach. Moreover, hazard ratios may be arduous to interpret by clinicians, as they provide a measure of therapeutic efficacy of a certain treatment only compared to an alternative without being informative on its absolute clinical benefit. In light of these considerations, after verifying the violation of the proportionality of hazards assumption for the reconstructed dataset derived from RCTs, we used an approach based on RMST to investigate differences in treatment response. OS, PFS and DoR at 18, 24 and 36 months were used as surrogate for therapeutic efficacy. In addition to a reduced risk of misinterpretation of the survival impact of different therapies, individual survival data methodology is the sole to allow for the evaluation of DoR, by reconstructing time-to-event data.

Despite its strengths, this study is not devoid of limitations. First, the examined populations, although similar in terms of demographics, local tumor management and IMDC risk groups, are not identical. Indeed, differences in geographic origin, metastatic burden or performance status may exist among RCT populations. These data were not available for all included studies and could thus not be considered. Moreover, differences in follow-up length between the included RCTs might also affect the oncological outcomes. Furthermore, meta-analysis methodology is unable to account for inter-population heterogeneity, randomized non-inferiority trials being the sole to minimize such biases. Therefore, the potential intrinsic heterogeneity in RCT populations warrants caution in interpreting results.

Second, the inherent diversity of single agents within the therapeutic class of IO-TKI combination (different IO classes and TKI agents) may lead to divergent treatment responses, which may remain undisclosed when all IO-TKI regimens are examined together. However, published RCTs on different TKI-IO combinations relative to sunitinib show comparable results.

Finally, lack of information on toxicity profiles also represent a potential study weakness. Indeed, clinicians should take into consideration safety, in addition to efficacy, when counselling mRCC patients.

## Conclusion

Herein, we reported a method that can be applied for meta-analyses in case of proportional hazard assumption violation. This method is particularly useful for analyzing data on duration of response. Using reconstructed survival data to estimate the survival impact of different therapies, we overcome biases associated with traditional meta-analysis methodologies and demonstrated that nivo-ipi and IO-TKI combinations are superior to sunitinib in terms of OS, PFS and DoR. The only significant difference between nivo-ipi and IO-TKI combinations was recorded in sustained DoR. Indeed, in addition to safety considerations and the presence of comorbidities, considering the shape of KM curves in spite of similar OS among IO-based strategies may aid clinicians be better informed for patient counselling and choosing tailored therapeutic options.

## Data availability statement

The original contributions presented in the study are included in the article/**Supplementary Material**, further inquiries can be directed to the corresponding authors.

## Author contributions

LN: Conceptualization, data curation, investigation, methodology, formal analysis, software, visualization, writing – original draft. GF: Data curation, investigation, methodology, formal analysis, software, visualization, writing – review and editing. DRa: Data curation, investigation, validation, writing – review and editing. FB: Data curation, investigation, validation, writing – review and editing. DRo: Data curation, investigation, validation, writing – review and editing. FM: Validation, writing – review and editing. PIK: Validation, writing - review and editing. BM: Validation, writing – review and editing. GP: Validation, writing – review and editing. AN: Conceptualization, validation, writing – review and editing, supervision. AM: Data curation, investigation, methodology, formal analysis, software, visualization, writing – original draft, supervision. All authors contributed to the article and approved the submitted version.

## Conflict of interest

Author AN: Consulting: Merck, Astra Zeneca, Janssen, Incyte, Roche, Rainier Therapeutics, Clovis Oncology, Bayer, and Astellas/Seattle Genetics, Ferring, Immunomedics. Grant/Research support: Merck, Ipsen, and Astra Zeneca. Travel expenses/Honoraria: Roche, Merck, Astra Zeneca, and Janssen. Authors AM, GF, GP and BM own equities of Oltre Medical Consulting, Toulouse, France.

The remaining authors declare that the research was conducted in the absence of any commercial or financial relationships that could be construed as a potential conflict of interest

## Publisher’s note

All claims expressed in this article are solely those of the authors and do not necessarily represent those of their affiliated organizations, or those of the publisher, the editors and the reviewers. Any product that may be evaluated in this article, or claim that may be made by its manufacturer, is not guaranteed or endorsed by the publisher.

## Supplementary material

Supplementary Figure 1 Overall survival of mRCC patients using reconstructed survival data derived from five individual studies with immune-oncology based combination therapy.

Supplementary Figure 2 Progression-free survival of mRCC patients using reconstructed survival data derived from five individual studies with immune-oncology based combination therapy.

Supplementary Figure 3 Duration of response of mRCC patients using reconstructed survival data derived from five individual studies with immune-oncology based combination therapy.

Supplementary Figure 4 Overall survival of intermediate/poor mRCC patients using reconstructed survival data derived from five individual studies with immune-oncology based combination therapy. Only data from CheckMate 214 and KEYNOTE-426 are included.

Supplementary Figure 5 Progression-free survival of intermediate/poor mRCC patients using reconstructed survival data derived from five individual studies with immune-oncology based combination therapy. Only data from CheckMate 214 and KEYNOTE-426 are included.
