# Supplementary material for: Immunotherapy in advanced kidney cancer: an alternative meta-analytic method using reconstructed survival data in case of proportional hazard assumption violation
Source: Front Oncol. 2022 Sep 5;12:955894. doi: 10.3389/fonc.2022.955894 (PMC9483094; doi:10.3389/fonc.2022.955894)

Supplementary Table 1. Assessment of risk of bias according to the Cochrane Risk of Bias tool for studies included in the meta-analysis.

Study	Random sequence generation (selection bias)	Allocation concealment (selection bias)	Blinding of participants and personnel (performance bias)	Blinding of outcome assessment (detection bias)	Incomplete outcome data (attrition bias)	Selective reporting (reporting bias)
CheckMate 214	Low risk	Unclear	At risk	At risk	Low risk	Low risk
KEYNOTE-426	Low risk	Unclear	At risk	Low risk	Low risk	Low risk
KEYNOTE-581	Low risk	Unclear	At risk	Low risk	Low risk	Low risk
JAVELIN Renal 101	Low risk	Unclear	At risk	Low risk	Low risk	Low risk
CheckMate 9ER	Low risk	Unclear	At risk	Low risk	Low risk	Low risk

Supplemental Table 2. Differences in restricted mean survival time (delta RMST) up to the time point of interest according to treatment arm.

Arms	ΔRMST up to 18mo of follow-up (95% CI)	ΔRMST up to 24mo of follow-up (95% CI)	ΔRMST up to 36mo of follow-up (95% CI)
	Overall survival		
Nivo-ipi vs sunitinib	1.1 (0.5 – 1.7)	1.8 (0.9 – 2.8)	3.2 (1.7 – 4.8)
Pembro-axi vs sunitinib	1.7 (1.1 – 2.3)	2.5 (1.6 – 3.5)	4.1 (2.4 – 5.7)
Nivo-ipi vs pembro-axi	-0.6 (-1.3 – 0.1)	-0.7 (-1.7 – 0.3)	-0.8 (-2.6 - 0.9)
	Progression-free survival		
Nivo-ipi vs sunitinib	1 (0.1 – 1.9)	1.7 (0.5 – 2.9)	3.6 (1.8 – 5.5)
Pembro-axi vs sunitinib	1.7 (0.8 – 2.9)	2.4 (1.2 – 3.7)	3.6 (1.7 – 5.3)
Nivo-ipi vs pembro-axi	-0.7 (-1.8 – 0.3)	-0.7 (-2.1 – 0.7)	0.02 (-2.2 – 2.2)

Supplemental Figure 1.

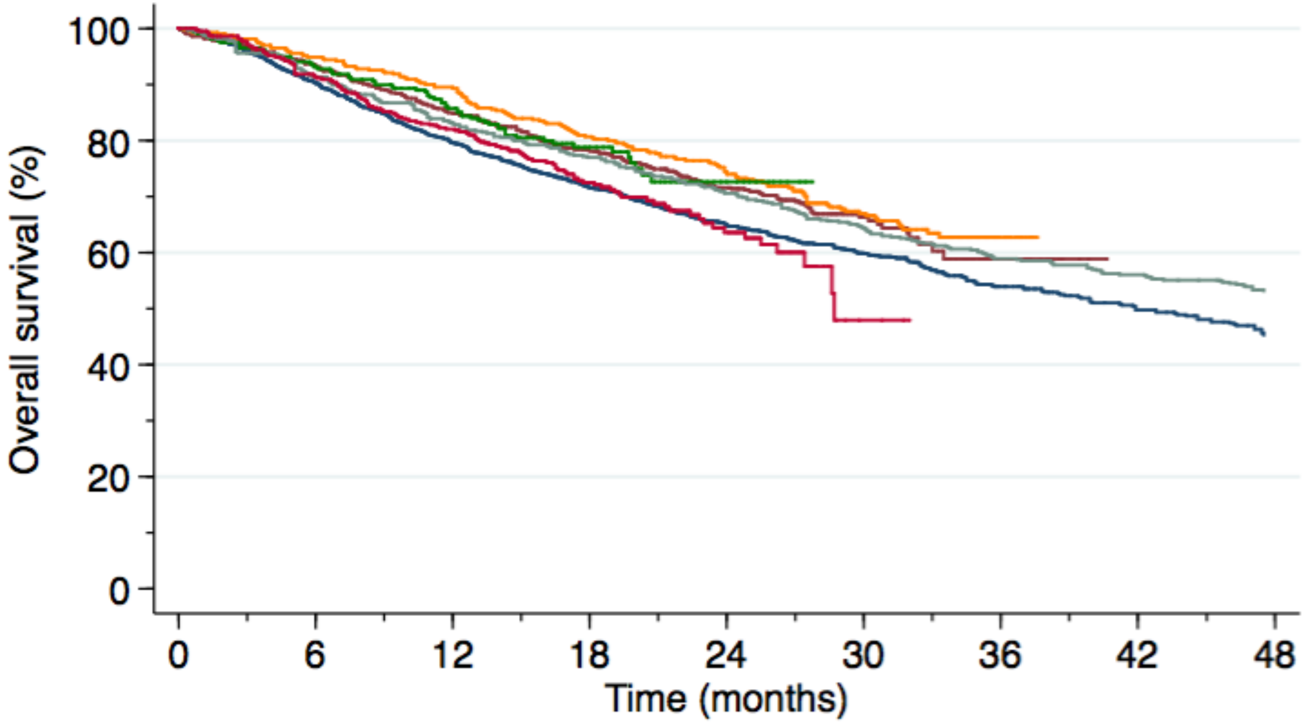

Number at risk

	0	6	12	18	24	30	36	42	48
Sunitinib	2102	1832	1588	1149	825	480	280	235	209
Pembro + Lenv	712	659	590	458	342	115	18	0	0
Pembro + Axi	432	408	385	346	305	163	23	0	0
Nivo + Cabo	323	295	259	111	13	0	0	0	0
Ipi + Nivo	550	493	444	411	372	337	307	292	271
Ave + Axi	444	387	338	186	74	4	0	0	0

Supplemental Figure 2.

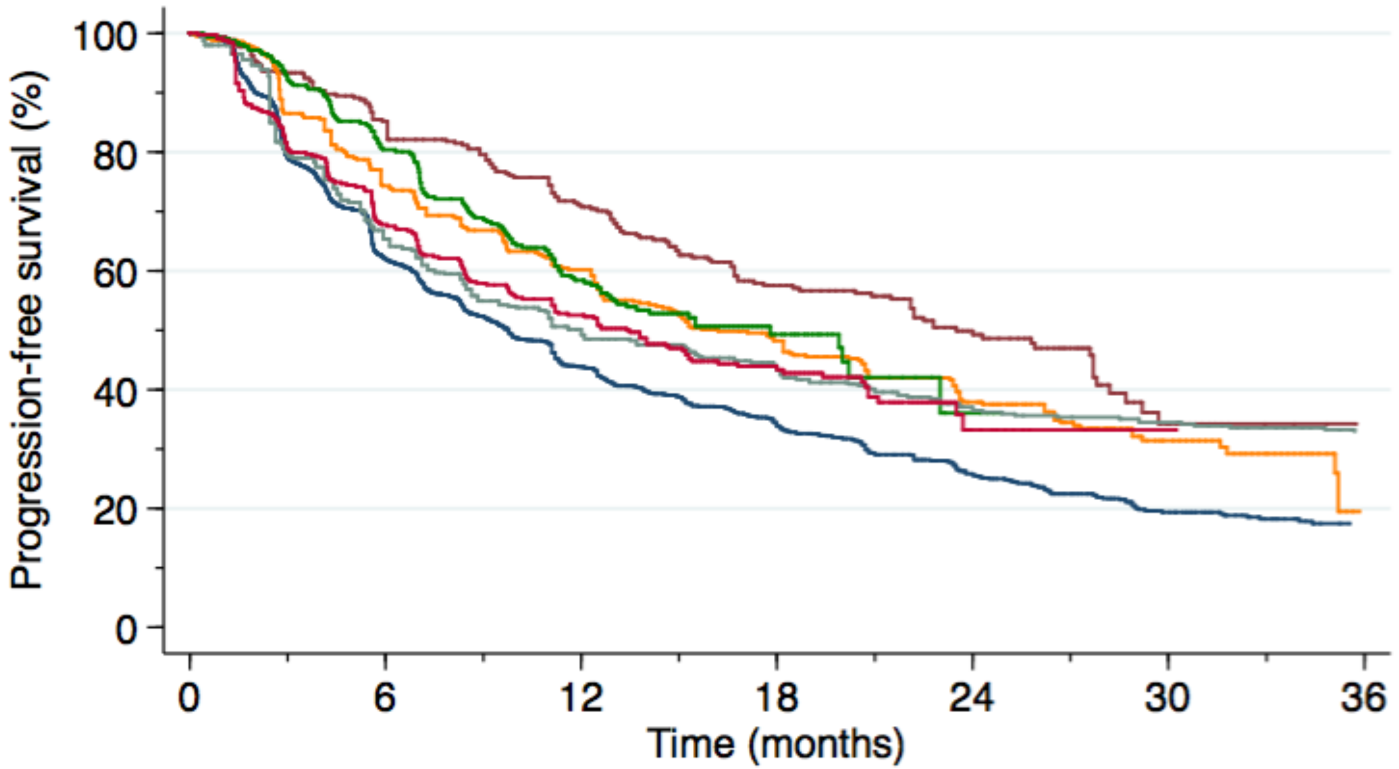

Number at risk

	0	6	12	18	24	30	36
Sunitinib	2104	1054	624	344	183	85	42
Pembro + Lenv	355	277	213	139	80	16	2
Pembro + Axi	432	300	234	180	109	37	2
Nivo + Cabo	323	234	144	35	4	0	0
Ipi + Nivo	550	315	215	170	130	118	99
Ave + Axi	442	264	194	79	10	1	0

Supplemental Figure 3.

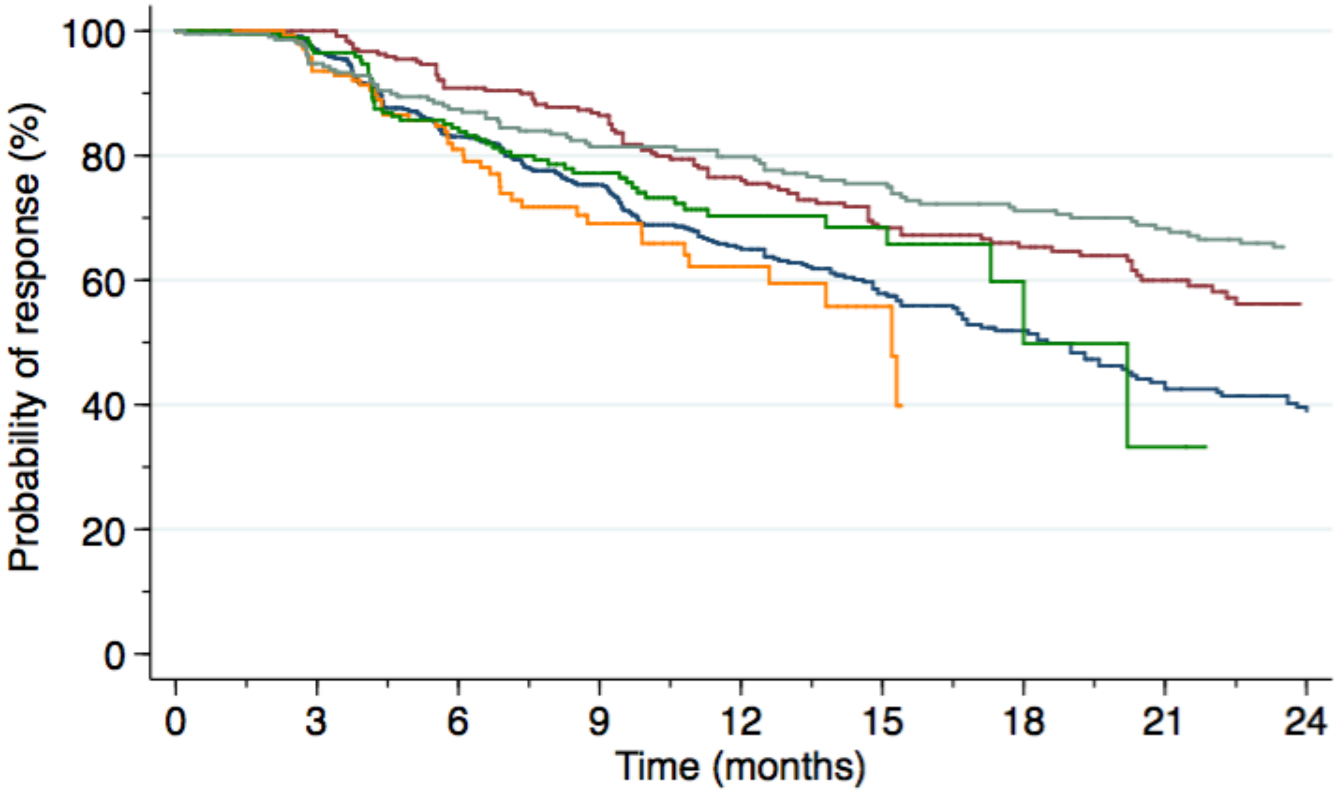

Number at risk

	0	3	6	9	12	15	18	21	24
Sunitinib	651	592	420	312	221	153	103	81	65
Pembro + Lenv	252	246	215	189	153	120	101	71	45
Pembro + Axi	153	130	84	49	26	9	0	0	0
Nivo + Cabo	180	164	135	106	58	25	6	2	0
Ipi + Nivo	215	197	176	158	149	139	128	118	108

Supplemental Figure 4.

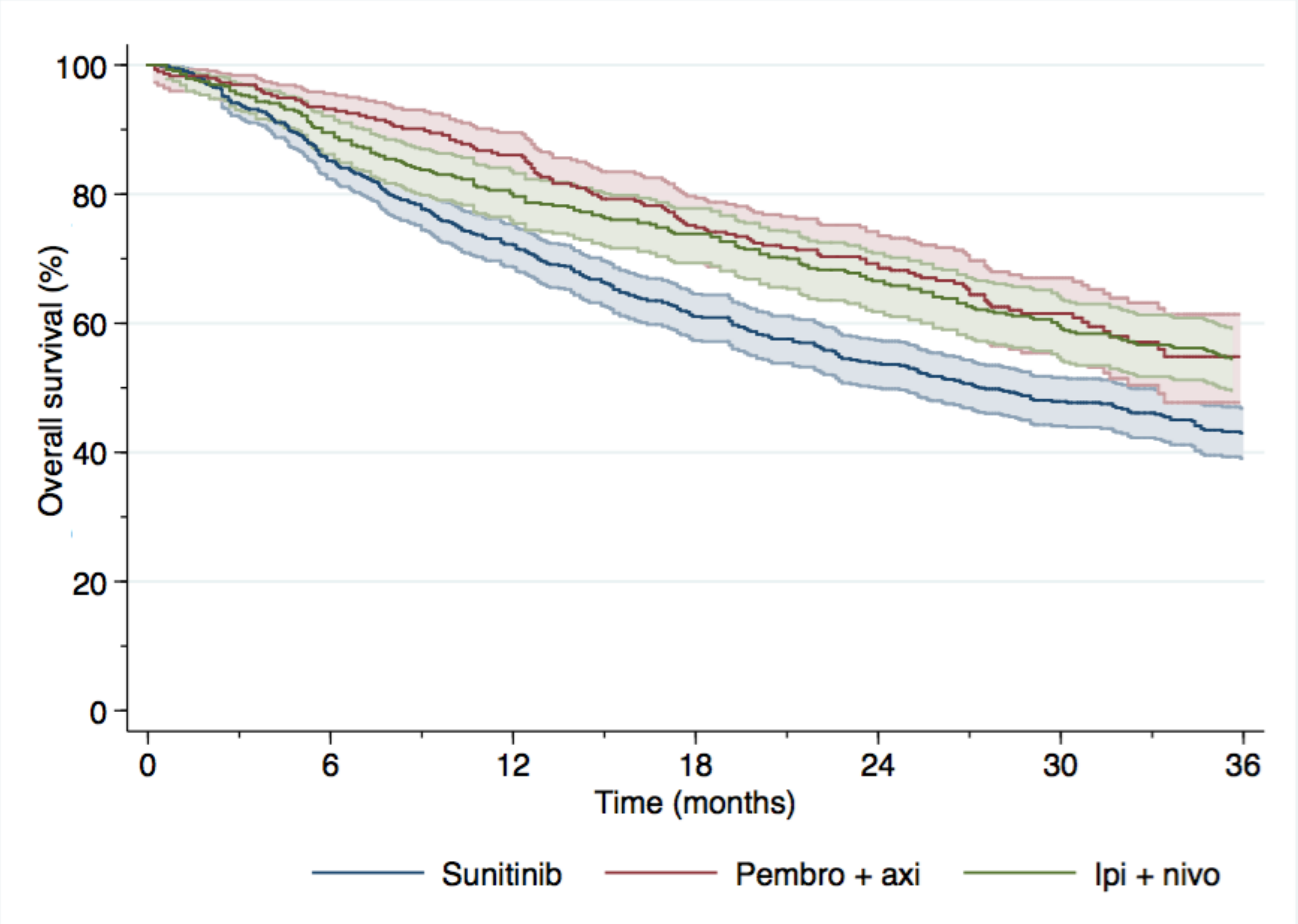

Supplemental Figure 5.

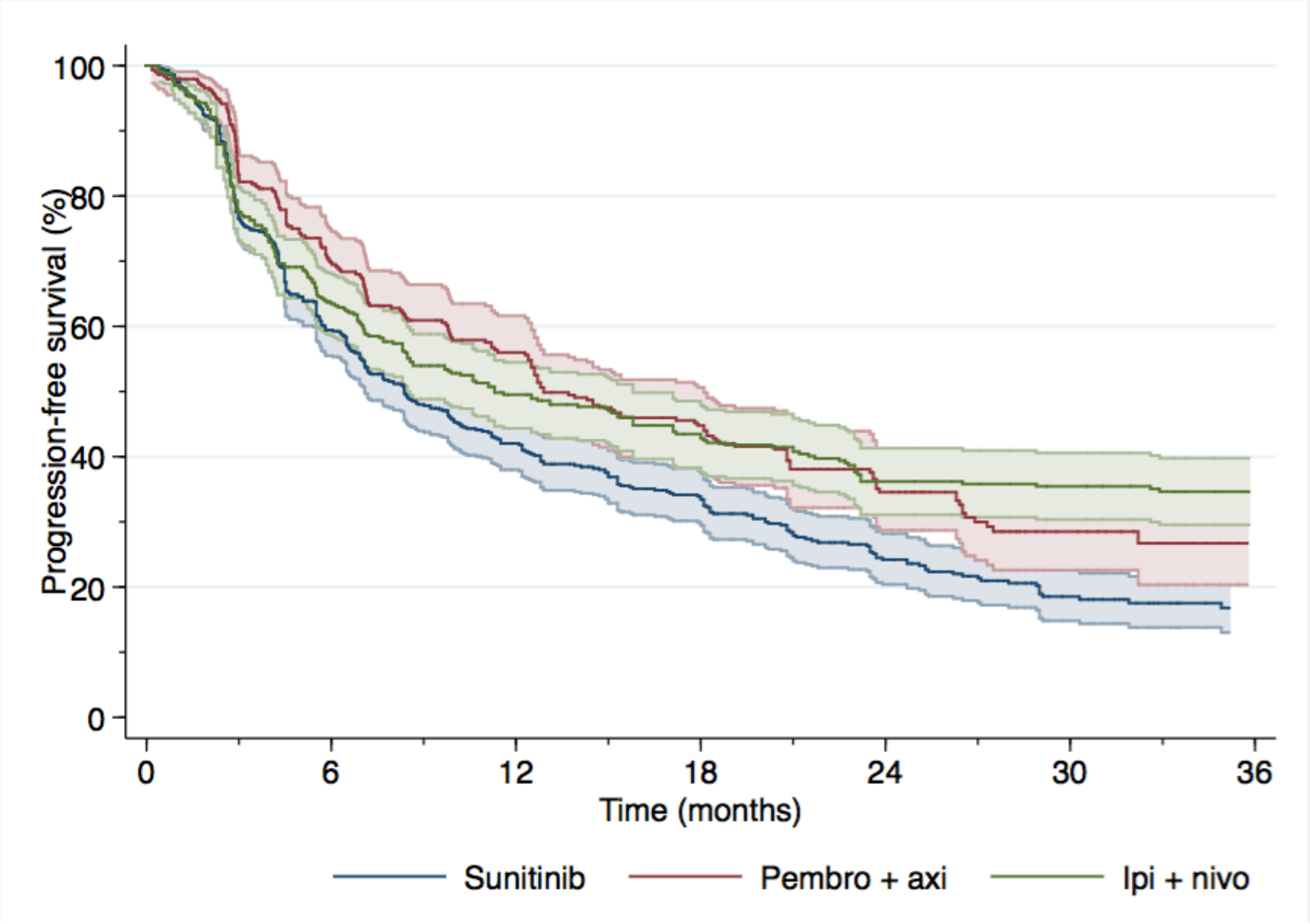

Supplement: Supplementary Figure 1 — Overall survival of mRCC patients using reconstructed survival data derived from five individual studies with immune-oncology based combination therapy. [file DataSheet_1.pdf]
